# Supplementary material for: Distinct Functional Alterations and Therapeutic Options of Two Pathological De Novo Variants of the T292 Residue of GABRA1 Identified in Children with Epileptic Encephalopathy and Neurodevelopmental Disorders
Source: Int J Mol Sci. 2022 Mar 1;23(5):2723. doi: 10.3390/ijms23052723 (PMC8911174; doi:10.3390/ijms23052723)
Supplement: Supplementary file 1 [file ijms-23-02723-s001.zip › ijms-1599563-supplementary.pdf]

International Journal of *Molecular Sciences*

MDPI

Article

Distinct Functional Alterations and Therapeutic Options of Two Pathological De Novo Variants of the T292 Residue of GABRA1 Identified in Children with Epileptic Encephalopathy and Neurodevelopmental Disorders

Supplementary Materials:

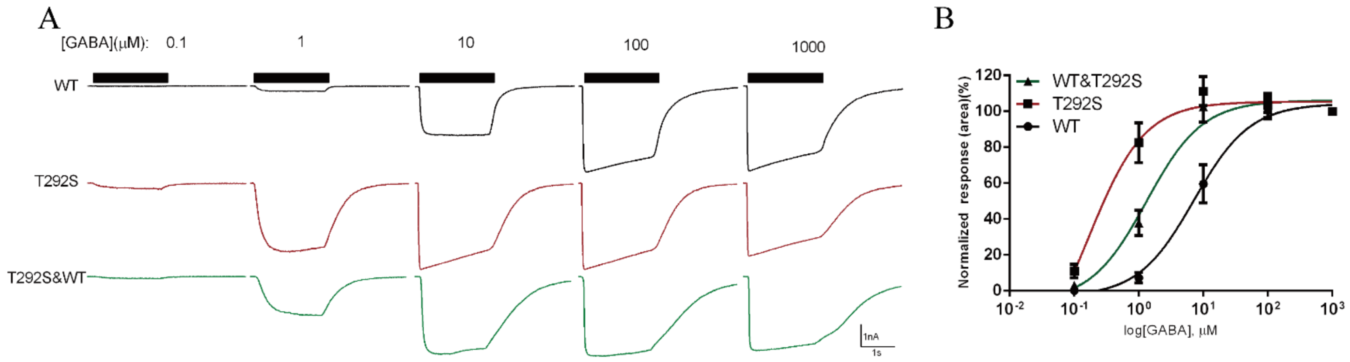

Figure S1. Overexpressing mixed GABRA1 subunits ($\alpha1_{T292}:\alpha1_{T292S}:\beta2:\gamma2$ = 1:1:2:1) in HEK293 cells to mimic heterozygous status in patient. **(A)** Representative trace of GABA-evoked currents of WT (black), $\alpha1_{T292S}$ GABA$_A$R (red) and mixed expression of $\alpha1_{T292}\alpha1_{T292S}\beta2\gamma2$ (green). **(B)** Dose-response curve of WT (n = 10), $\alpha1_{T292S}$ GABA$_A$R (n = 8) and mixed expression of $\alpha1_{T292}\alpha1_{T292S}\beta2\gamma2$ (n = 8).

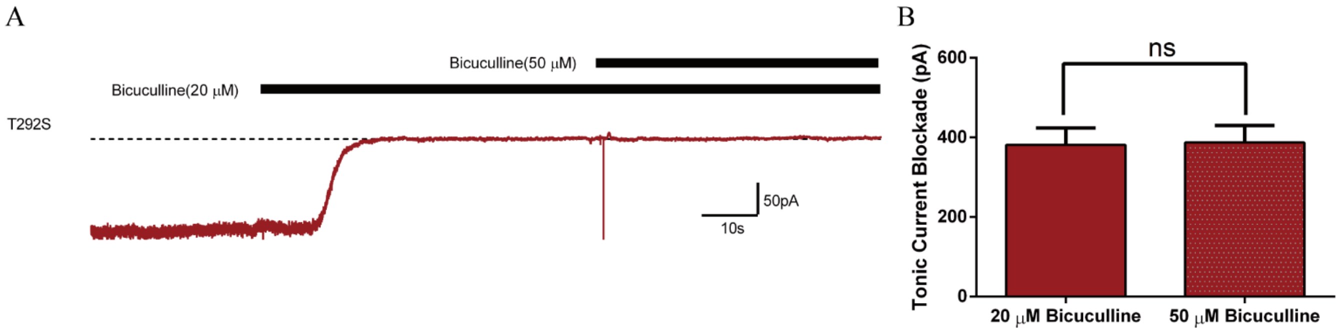

Figure S2. Higher concentration bicuculline could not further block tonic current of α1T292S GABA$_A$R **(A)** Representative trace of 20 μM and 50 μM bicuculline blocked tonic current of $\alpha1_{T292S}$ GABA$_A$R. **(B)** Quantification of tonic current blocked by 20 μM and 50 μM bicuculline of $\alpha1_{T292s}$ (n = 7) of GABA$_A$R. Statistical differences were determined using unpaired t-test (ns = not significant).

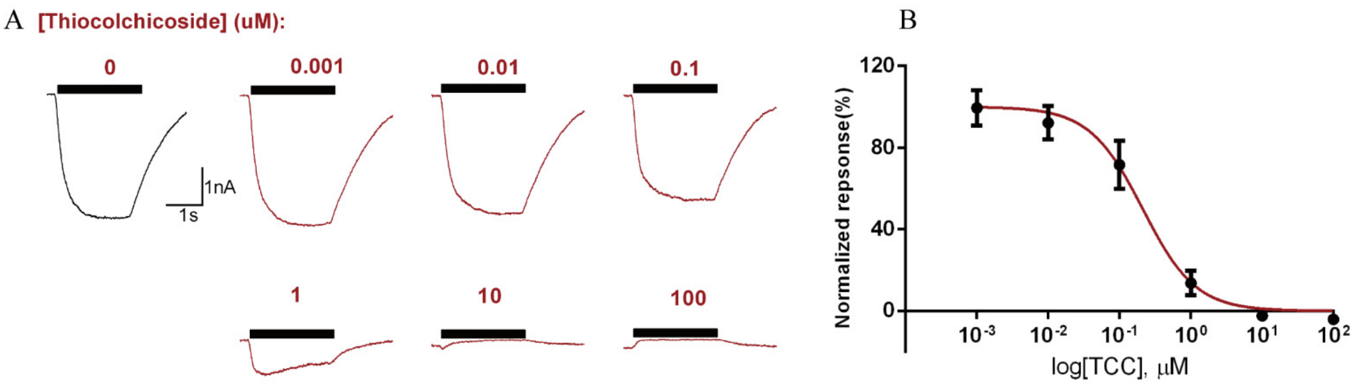

Figure S3. (A) Representative trace and **(B)** dose-response curve of TCC on 1μM GABA-evoked current to determine the optimal concentration of thiocolchicoside to restore the GABA-evoked currents of T292S GABAARs to the wild-type level.
